# Complete Genome Sequence of the Bovine Mastitis Pathogen *Mycoplasma californicum* Strain ST-6^T^ (ATCC 33461^T^)

**DOI:** 10.1128/genomeA.00648-14

**Published:** 2014-07-03

**Authors:** Michael J. Calcutt, Mark F. Foecking, Lawrence K. Fox

**Affiliations:** a Department of Veterinary Pathobiology, University of Missouri, Columbia, Missouri, USA; b Department of Veterinary Clinical Sciences, Washington State University, Pullman, Washington, USA

## Abstract

*Mycoplasma californicum* is one of several mycoplasmal species associated with bovine mastitis. The complete genome sequence of 793,841 bp has been determined and annotated for the *M. californicum* ST-6 type strain, providing a resource for the identification of surface antigens and putative pathoadaptive features.

## GENOME ANNOUNCEMENT

Mycoplasma mastitis is an increasing problem for the U.S. dairy industry (1). *Mycoplasma bovis* is the most commonly isolated species from the group of wall-less pathogens associated with mastitis, with *Mycoplasma californicum* being the second most prevalent in some studies (2, 3). In contrast to *M. bovis*, almost nothing is known about the gene portfolio and virulence factors of *M. californicum*. To address these knowledge gaps, the genome sequence of the type strain, first isolated from a mastitis outbreak in California in 1972 (4), was determined and annotated.

Genomic DNA was prepared from *M. californicum* ST-6^T^ (obtained from the International Organization for Mycoplasmology [IOM] culture collection, Purdue, IN) and sequenced using the Pacific Biosciences platform at the National Center for Genome Resources (NCGR), Santa Fe, NM. A single SMRT cell was used to generate 181,998 reads that were assembled using HGAP version 2 (5). As a result, 1 large (~790 kb) and two small (~3.8 kb) contigs emerged, with 230× coverage, which were readily closed by PCR and Sanger sequencing. The 793,841-bp genome sequence was automatically annotated using the PGAP pipeline at NCBI, following which the resulting open reading frames (ORFs) were manually curated. The genome comprises 669 genes with 611 ORFs, 21 independently verified pseudogenes, 31 tRNAs, and two copies of each rRNA (with 5S rRNA genes separated from the two 16S-23S rRNA operons). The G+C content is 30.84%.

A motif query for surface lipoproteins disclosed 52 putative lipoprotein-encoding genes, including genes for two tandem paralogs of P30 (an immunodominant antigen of *Mycoplasma agalactiae* [6]) and two paralogs of MALP-404 (7). The presence of lipoprotein genes encoding nucleases (three predicted), acid phosphatase, and 5′-nucleotidase, together with a homolog of the polynucleotide binding lipoprotein (8), highlights the critical importance of nucleotide acquisition in the absence of complete nucleotide biosynthesis pathways.

The ability of mycoplasmas to undergo high-frequency switching of surface antigens has long been recognized (9) and is predicted to reflect successful adaptation to the host (10). Interrogation of the genome for distinctive hypermutable motifs revealed that the ORFs for 7 surface lipoproteins were preceded by perfect (6 instances) or near-perfect homopolymeric tracts that potentially orchestrate phase-variable expression resulting in combinatorial surface protein arrays (11).

Mycoplasmas are amino acid auxotrophs, necessitating the acquisition of such precursors by proteolysis and transport (12). *M. californicum* and the most closely related organism *Mycoplasma bovigenitalium* (13) are unusual among *Mycoplasma* species in that both possess a gene encoding the tryptophan synthase beta-subunit (TrpB). In most organisms that contain TrpB, this protein functions as an α_2_β_2_ tetramer with TrpA (14). This multimer is the best studied example of substrate channeling (15); TrpA cleaves indole glycerol 3-phosphate to glyceraldehyde 3-phosphate and indole, with indole passing directly through an interconnecting tunnel for tryptophan synthesis by TrpB. An orphan *trpB* gene is unusual in prokaryotes and raises the possibility that the organism can synthesize tryptophan from exogenous indole.

The annotated genomic sequence is the first for this species and expands the data sets available for studying the evolution and host adaptation of ruminant mycoplasmas.

### Nucleotide sequence accession number.

This complete genome sequence has been deposited at DDBJ/EMBL/GenBank under the accession no. CP007521.
